# Transducin-like enhancer of split 3 regulates proliferation of melanoma cells via histone deacetylase activity

**DOI:** 10.18632/oncotarget.26552

**Published:** 2019-01-08

**Authors:** Masahiro Ogawa, Tatsuki Yaginuma, Chihiro Nakatomi, Tsuyoshi Nakajima, Yukiyo Tada-Shigeyama, William N. Addison, Mariko Urata, Takuma Matsubara, Koji Watanabe, Kou Matsuo, Tsuyoshi Sato, Hiromi Honda, Hisako Hikiji, Seiji Watanabe, Shoichiro Kokabu

**Affiliations:** ^1^ Division of Molecular Signaling and Biochemistry, Department of Health Improvement, Kyushu Dental University, Kitakyushu, Fukuoka, Japan; ^2^ Division of Dental Anesthesiology, Department of Science of Physical Functions, Kyushu Dental University, Kitakyushu, Fukuoka, Japan; ^3^ Research Unit, Shriners Hospitals for Children-Canada, Department of Human Genetics, McGill University, Montreal, Quebec, Canada; ^4^ Division of Developmental Stomatognathic Function Science, Department of Health Improvement, Kyushu Dental University, Kitakyushu, Fukuoka, Japan; ^5^ Division of Oral Pathology, Department of Health Improvement, Kyushu Dental University, Kitakyushu, Fukuoka, Japan; ^6^ Department of Oral and Maxillofacial Surgery, Faculty of Medicine, Saitama Medical University, Moroyama-machi, Iruma-gun, Saitama, Japan; ^7^ School of Oral Health Sciences, Kyushu Dental University, Kitakyushu, Fukuoka, Japan

**Keywords:** malignant melanoma, transcriptional co-repressor, trichostatin A, HDAC inhibitors

## Abstract

Melanoma, one of the most aggressive neoplasms, is characterized by rapid cell proliferation. Transducin-like Enhancer of Split (TLE) is an important regulator of cell proliferation via Histone deacetylase (HDAC) recruitment. Given that HDAC activity is associated with melanoma progression, we examined the relationship between TLE3, a TLE family member, and melanoma. TLE3 expression was increased during the progression of human patient melanoma (p < 0.05). Overexpression of Tle3 in B16 murine melanoma cells led to an increase in cell proliferation (p < 0.01) as well as the number of cyclinD1-positive cells. *in vivo* injection of mice with B16 cells overexpressing Tle3 resulted in larger tumor formation than in mice injected with control cells (p < 0.05). In contrast, siRNA-mediated knockdown of Tle3 in B16 cells or TLE3 in HMV-II human melanoma cells decreased proliferation (p < 0.01). Treatment of B16 cells with trichostatin A (2.5 μM), a class I and II HDAC inhibitor, prevented the effect s of Tle3 on proliferation. In conclusion, these data indicate that Tle3 is required, at least in part, for proliferation in the B16 mouse melanoma model.

## INTRODUCTION

Malignant melanoma is one of the most aggressive neoplasms. The worldwide incidence of melanoma has been steadily increasing with mortality rates rising faster than any other form of cancer [1]. Melanoma cells are derived from the neural crest and characterized by rapid proliferation and numerous distal metastasis [2]. Recently, major advancements have been achieved for metastatic melanomas via the blockade of immune-checkpoints using a programmed death 1 (PD-1) checkpoint inhibitor and a cytotoxic T-lymphocyte-associated antigen 4 (CTLA-4) checkpoint inhibitor [3]. However, these drugs may cause adverse immune-related events such as interstitial pneumonia, large intestine inflammation, and type I diabetes [4]. Furthermore, primary non-response as well as acquired resistance to immune-checkpoint blockers remain a challenge. Thus, the need for the development of novel treatment approaches remain.

Histone deacetylases (HDACs) remove acetyl groups from lysine residues on histones. Removing the acetyl group alters chromatin structure by facilitating chromatin condensation to promote transcriptional repression [5]. Aberrant HDAC expression, dis-regulation of HDAC activity or imbalances between HDACs and histone acetyltransferases are likely involved in the development and progression of several malignant tumors [6]. Melanomas contain high levels of HDAC-1, 2 and melanoma cells have been shown to overexpress HDAC-1, -2, -3, compared to non-malignant cells [6]. HDAC inhibitors are pharmacologic compounds that interfere with the deacetylation reaction mediated by HDACs. HDAC inhibitors have potent anti-proliferative effects on melanoma cells [7–9] and thus represent promising therapeutic agents for malignant melanoma [10].

Transducin-like Enhancer of Split (TLE) family members are transcriptional co-factors that play critical roles in cell proliferation and differentiation [11]. Recently, we reported that TLE3, a TLE family member, induces cell proliferation and suppresses cell differentiation skeletal muscle stem cells. Skeletal muscle stem cells, like melanocytes, are also mesenchymal lineage cells [12]. TLE proteins do not bind DNA directly but are instead recruited to chromatin by other transcription factors where they then reduce the activity of a target transcriptional factor [11]. TLE proteins consist of a five-domain structure [13]: Q domain; a glycine/proline rich (GP) domain; a CcN domain; a serine/proline rich (SP) domain; and a WD40 domain. TLE proteins usually work as transcriptional co-repressors by interacting with and recruiting HDACs [14]. The GP domain is especially essential for interaction of TLE proteins with HDACs [15–18].

Here, we examined the relationship between expression of TLE3 and malignant melanoma, as well as the effect of TLE3 on cell proliferation using melanoma cells.

## RESULTS

### TLE3 expression levels are increased in human malignant melanomas

**Figure 1:**
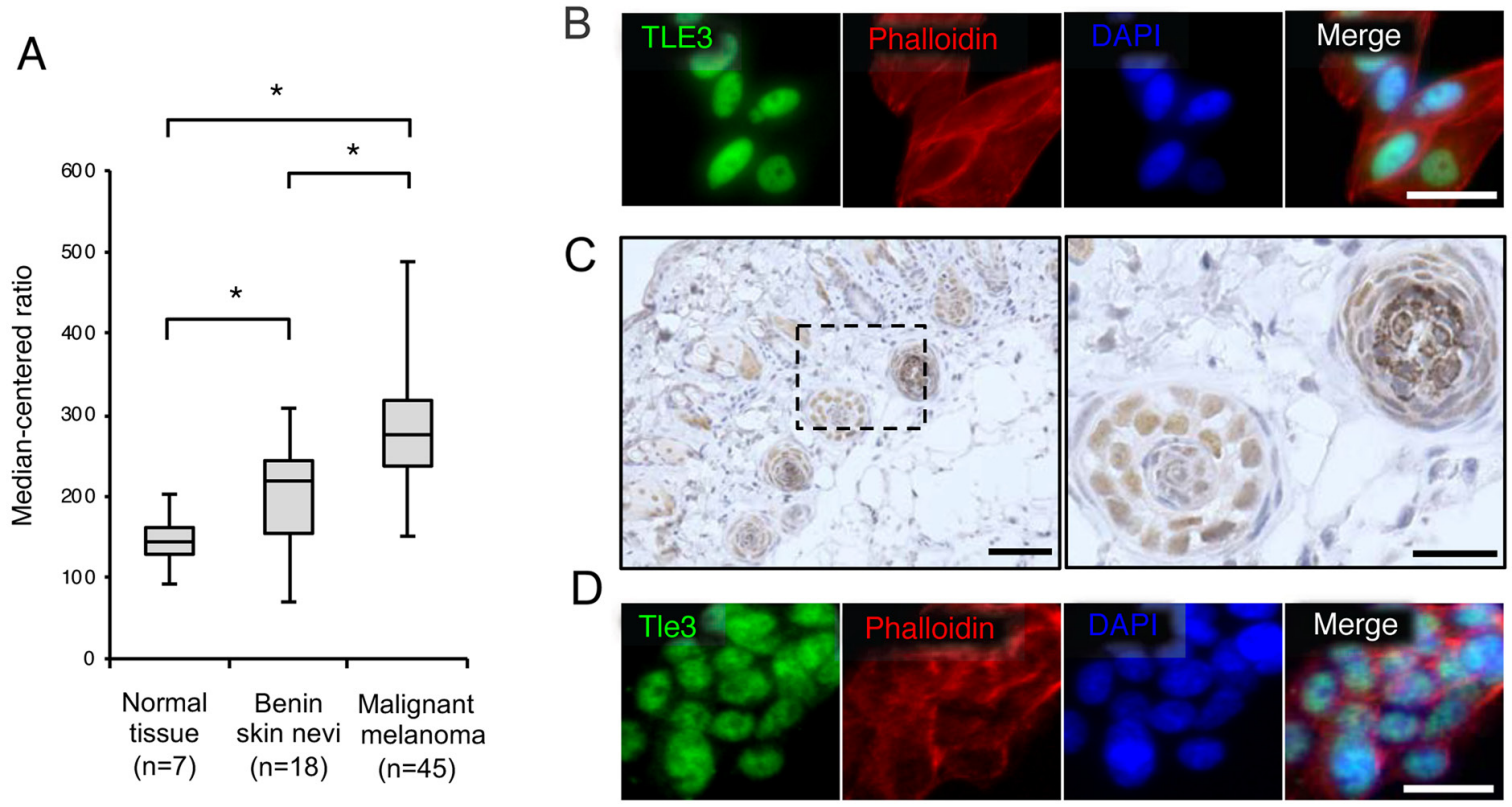
The expression levels of TLE3 are increased in human malignant melanoma The expression of TLE3 in normal skin, benign nevi, and malignant melanoma of patients (GSE3189 dataset) [19]. Expression levels of TLE3 are presented as boxplots and means were compared using unpaired ANOVA with Tukey-Kramer post-hoc test and Wilcoxon’s signed rank test **(A)**. HMV-II cells were stained with TLE3 antibody, rhodamine phalloidin (phalloidin), or DAPI **(B)**. Skin from 12-week-old C57BL/6J male mice was immunostained with anti-Tle3 antibody. The boxed areas in the left panel are shown as magnified images of hair follicles in the right panel. Scale bars indicate 500 μm (left panel) and 100 μm (right panel) respectively. Representative images of several sections are shown **(C)**. B16 cells were stained with Tle3 antibody, phalloidin, or DAPI **(D)**. Representative images are several experimental repeats shown. Scale bar corresponds to 100 μm (B and D).

We first examined the mRNA levels of *TLE3* in human melanoma patients by analyzing an NCBI Gene Expression Omnibus (GEO) dataset of melanoma microarray profiles [19]. The expression of *TLE3* in benign skin nevi was higher than in normal skin. The expression of *TLE3* was further increased in malignant samples compared to benign skin nevi (Figure 1A), suggesting that the expression of TLE3 is involved in the progression of melanoma. We then confirmed whether TLE3 was expressed in an additional melanoma cell type. Immunofluorescence imaging revealed that TLE3 was also expressed in HMV-II human melanoma cells (Figure 1B). next, we examined Tle3 expression in murine melanocytes. Tle3 was highly expressed in hair follicles melanocytes, which contain distinct melanin granules (Figure 1C and Supplementary Figure 1). We also confirmed that Tle3 was expressed within the nuclei of B16 murine melanoma cells (Figure 1D).

### Overexpression of Tle3 increases the proliferation of B16 melanoma cells

**Figure 2:**
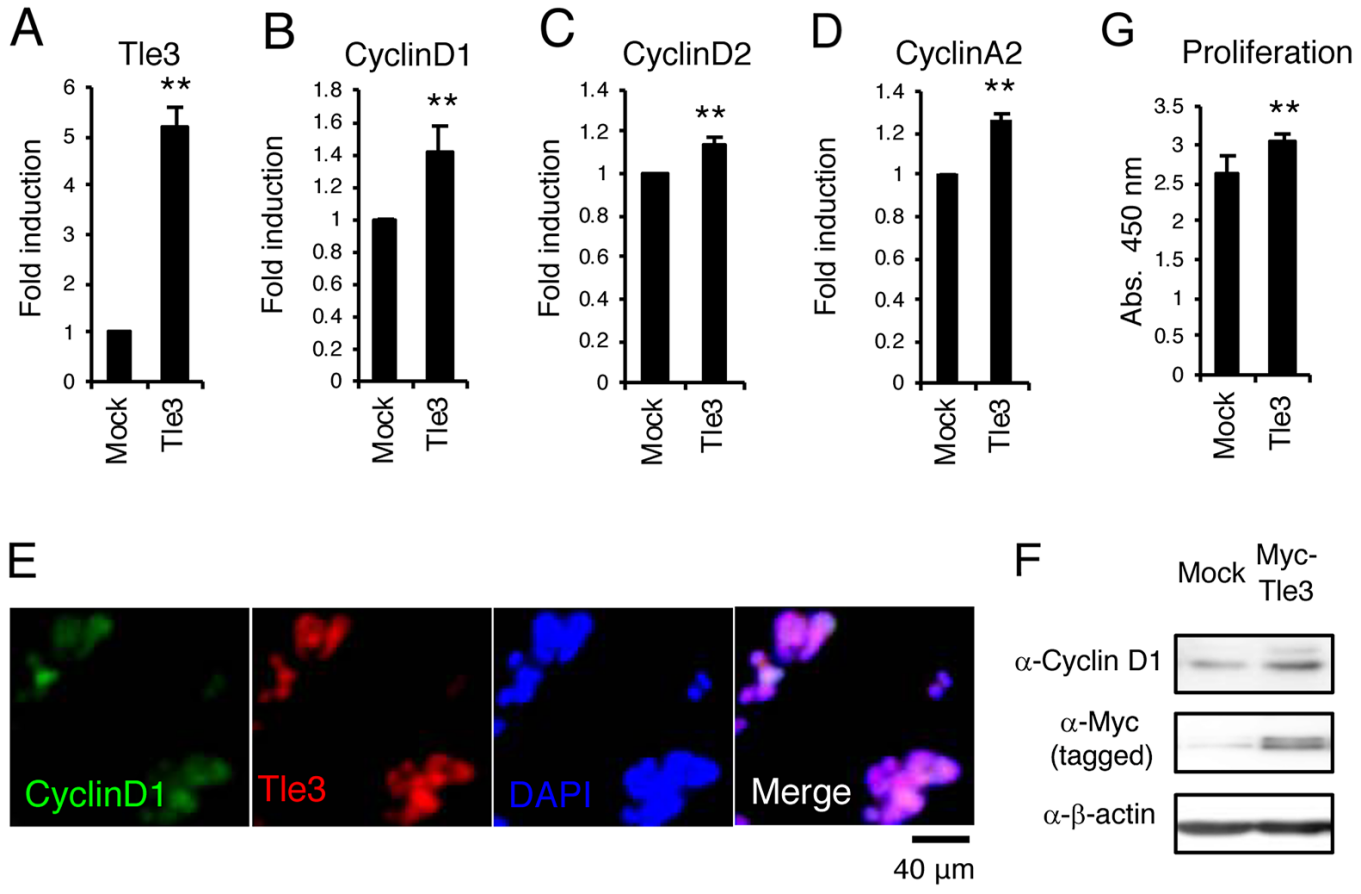
Overexpression of Tle3 increases proliferation in B16 melanoma cells **(A**-**F)** B16 cells stably expressing Myc-tagged Tle3 or empty vector were generated after positive selection with G418. The messenger RNA levels of Tle3 (A), CyclinD1 (B), CyclinD2 (C), or CyclinA2 (D) were determined by qPCR on day 2. B16 cells with high expression of Tle3 co-expressed CyclinD1 in the nuclei. Scale bar corresponds to 40 μm (E). Protein levels of CyclinD1, Myc-tagged Tle3, or β-actin were determined by western-blot analysis on day 2 (F). Overexpression of Tle3 increased the proliferation of B16 cells assessed by water-soluble tetrazolium salt (WST) -8 assay. Proliferation was quantified on day 2 by spectrophotometric absorbance measurement at 450 nm **(G)**. Data are expressed as the mean ± SD (n = 3). ^**^, p < 0.01 versus control (A-D, G). Representative images were shown (E and F).

**Figure 3:**
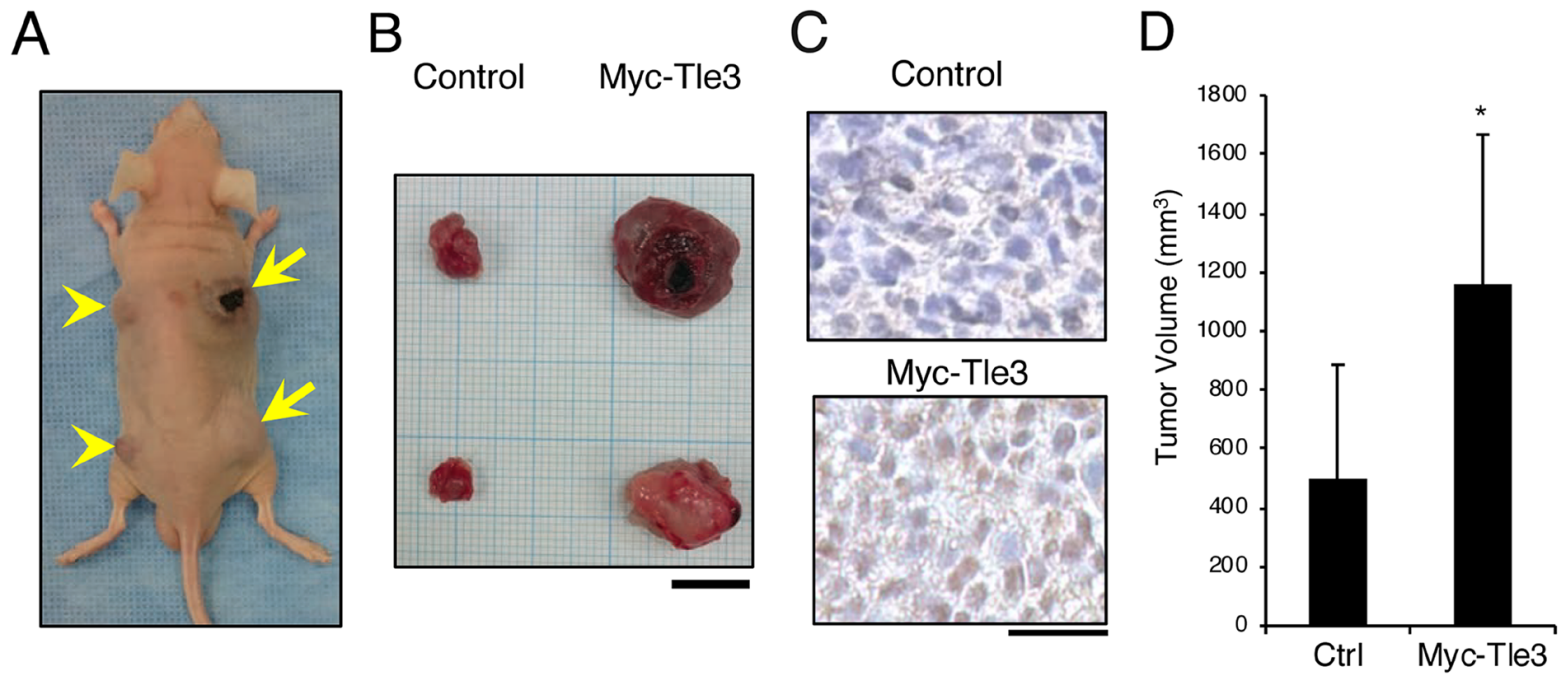
Overexpression of Myc-Tle3 in subcutaneously injected B16 melanoma cells increases tumor size *in vivo* BALB/cA Jcl-nu/nu mice (n=5) were injected subcutaneously with 1 × 10^5^ control B16 cells (left side; arrow heads) or cells stably expressed Myc-tagged Tle3 (right side; arrows). Representative photograph of a mouse **(A)** and resected tumors **(B)** 3 weeks after injection of with B16 cells. Scale bar corresponds to 10 mm (B). Resected tumors were immunostained with anti-Tle3 antibody. Scale bars indicate 25 μm **(C)**. Representative images of several sections are shown. The volume of resected tumors was quantified. The data are expressed as the mean ± SD (n = 10). ^*^, p < 0.05 versus control **(D)**.

A characteristic feature of melanoma is rapid cell proliferation [2]. Tle3 has been shown to stimulate cell proliferation in skeletal muscle satellite cells [12]. We hypothesized that Tle3 may also play role in proliferation of melanoma cells. Overexpression of Tle3 in B16 melanoma cells stimulated mRNA expression of cell cycle related genes such as *CyclinD1*,*CyclinD2*, and *CyclinA2* (Figure 2B-2D). Immunofluorescence staining showed that CyclinD1 expression correlated with the overexpression of Tle3 (Figure 2E). Consistent with the changes in mRNA expression, overexpression of TLE3 also increased the protein levels of CyclinD1 (Figure 2F). An *in vitro* cell proliferation assay also demonstrated that overexpression of Tle3 increased the proliferation of B16 cells (Figure 2G). To determine if Tle3 affects proliferation *in vivo*, we subcutaneously injected mice with B16 cells over-expressing Tle3 and assessed the development of tumors. The size of tumors formed from B16 cells over-expressing Tle3 was larger than from control cell tumors (Figure 3). These data indicate that Tle3 stimulates cell proliferation of B16 *in vivo* and *in vitro*.

### Knockdown of Tle3 (TLE3) in melanoma cells decreases proliferation

**Figure 4:**
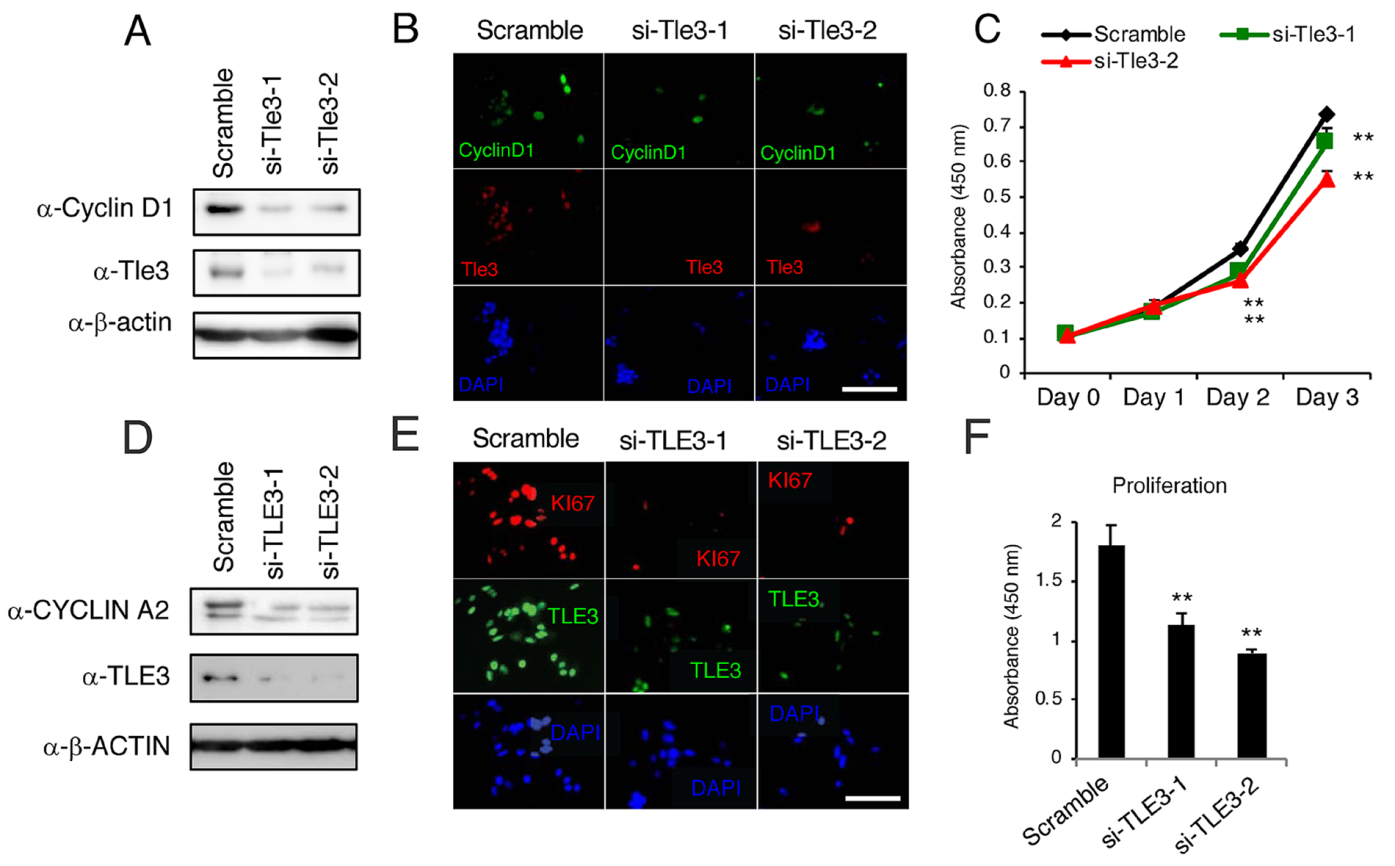
Knockdown of Tle3 (TLE3) in melanoma cells decreases proliferation **(A-C)** B16 cells were transfected with scramble siRNA, or siRNA against murine Tle3 (siTle3-1, siTle3-2). Protein levels of Tle3, cyclinD1, or β-actin were assessed by western blotting analysis (A). The numbers of cyclinD1 positive cells were decreased in the Tle3 knockdown B16 cells (B). In cells Tle3 knockdown cells, proliferation ability on day 2 and day 3 was decreased in comparison to scramble siRNA cells (C). **(D-F)** HMV-II cells were transfected with scrambled siRNA or siRNA against human TLE3 (siTLE3-1, siTLE3-2). Protein levels of TLE3, CYCLIN A2, or β-ACTIN were assessed by western blotting analysis (D). The numbers of KI67 positive cells were decreased in the TLE3 knockdown HMV-II cells (E). In TLE3 knockdown HMV-II cells, proliferation on day 4 was decreased in comparison to scrambled siRNA cells (F). Scale bar corresponds to 100 mm (B and E). ^**^, p < 0.01 versus scramble (C and F).

**Figure 5:**
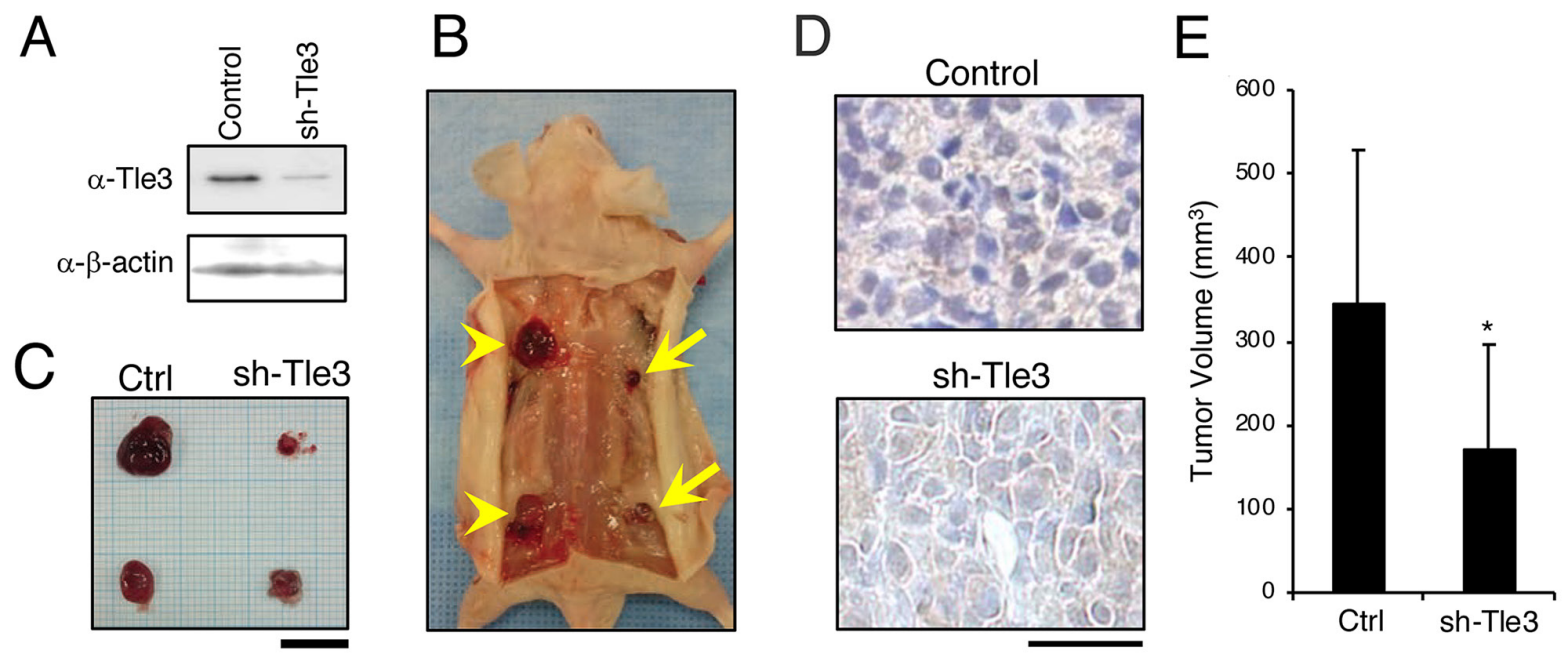
Knockdown of Tle3 in subcutaneously injected B16 melanoma cells decreases tumor size *in vivo* B16 cells, stably expressing control shRNA (Control) or shRNA against Tle3 (sh-Tle3) were generated by blasticidin selection. Protein levels of Tle3 were decreased in cells stably expressing sh-Tle3 **(A)**. BALB/cA Jcl-nu/nu mice (n=5) were injected subcutaneously with 1 × 10^5^ control cells (left side; arrow heads) or sh-Tle3 (right side; arrows). Representative pictures of subcutaneous tumors **(B)** and resected tumors **(C)** 3 weeks after injection of B16 cells are shown. Scale bar corresponds to 10 mm (C). Resected tumors were immunostained with anti -Tle3 antibody. Scale bars indicate 25 μm. Representative images of several sections are shown **(D)**. The volume of resected tumors were quantified (E). The data are expressed as the mean ± SD (n = 10). ^*^, p < 0.05 versus control **(E)**.

We next examined the effect of Tle3 reduction on the proliferation of B16 cells. In contrast to the effect of Tle3 overexpression, siRNA-mediated knockdown of Tle3 led to a reduction in the protein levels of CyclinD1 as well as a reduction in the number of CyclinD1 positive cells (Figure 4A and 4B). An *in vitro* cell proliferation assay also demonstrated that Tle3 knockdown cells proliferated at a reduced rate compared to Control siRNA cells (Figure 4C). In human HMV-II melanoma cells, siRNA knockdown of TLE3 expression resulted in a reduction of CYCLIN A2 protein levels (Figure 4D). siRNA-mediated knockdown of TLE3 led to a reduction in the number of KI67-positive cells (Figure 4E) as well as a decrease in proliferation (Figure 4F) compared to Control siRNA cells. Moreover, the size of tumors derived from the subcutaneous injection of B16 cells in which *Tle3* had stably been knocked-down by shRNA were smaller than that of control tumors (Figure 5). These data indicate that Tle3 is required, at least in part, for proliferation in the B16 mouse melanoma model.

### TLE3 induces proliferation of B16 cells via HDACs

**Figure 6:**
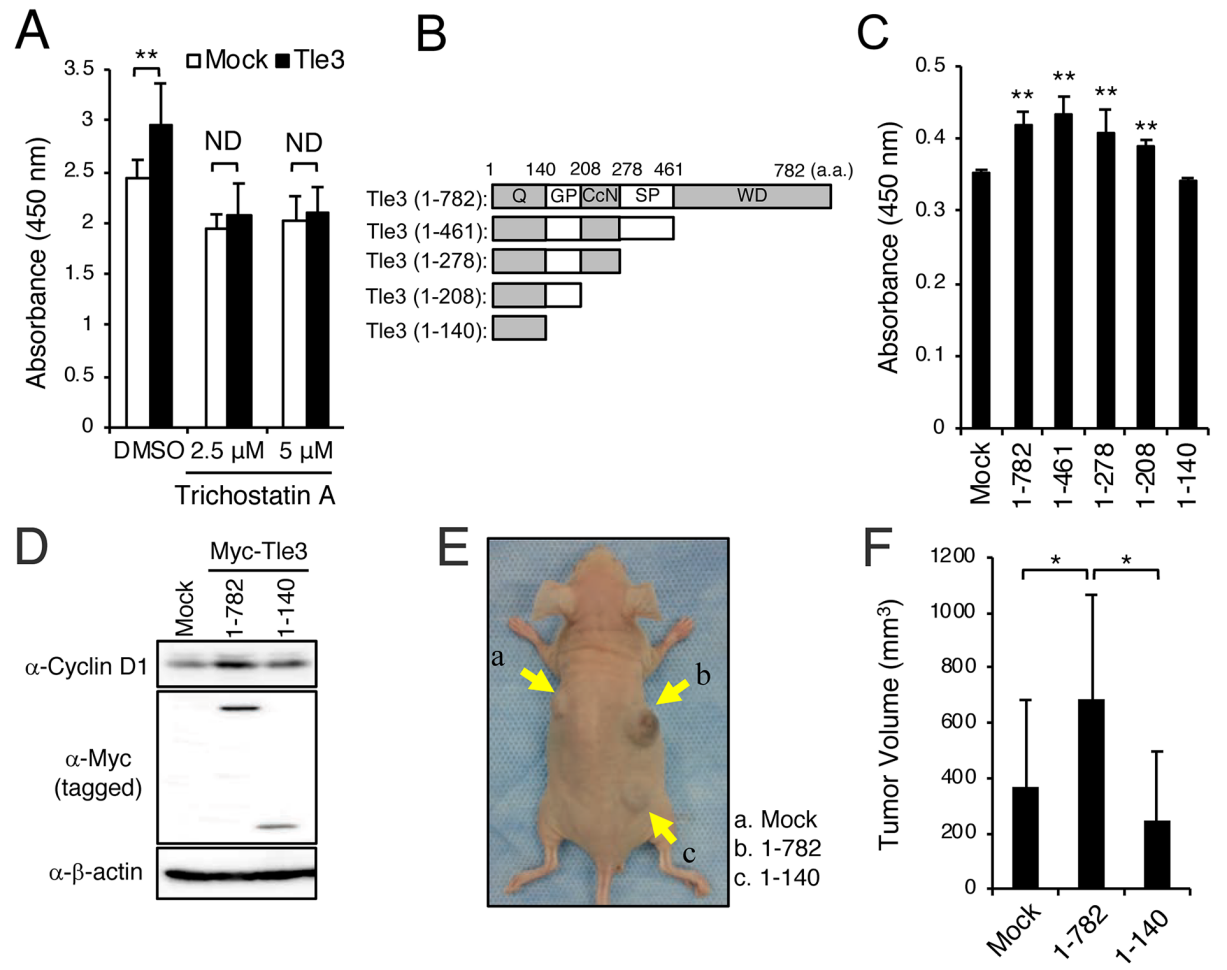
HDACs are involved in the enhancement of the proliferation of B16 cells by Tle3 **(A-C)** B16 cells were transiently transfected with empty vector (Mock) or Myc-tagged Tle3 and then treated with DMSO, or the indicated concentration of trichostatin A. Cell proliferation was evaluated on day 2 by water-soluble tetrazolium salt (WST) assay and absorbance measurement at 450 nm (A). Schematic of the C-terminally truncated forms of the Myc-tagged Tle3 plasmids used in these experiments. Q; glutamine rich domain, GP; glycine/proline rich domain, CcN; CcN domain, SP; serine/proline rich domain, WD, tryptophan/aspartic acid repeat domain (B). C-terminally truncated forms of Tle3 were transfected in B16 cells and proliferation ability measured on day 2 by WST assay (C). The data are expressed as the mean ± SD (n = 3). ^**^, p < 0.01 versus Mock transfection (A and C). B16 cells were transfected with empty vector (Mock), Myc-tagged Tle3 (1-782), or Myc-tagged Tle3 (1-140). Protein levels of cyclinD1, Myc (tagged) or β-actin were assessed by western blotting analysis on day 2 **(D)**. **(E and F)** B16 cells stably expressing Myc-tagged Tle3 (1-782), Myc-tagged Tle3 (1-140), or empty vector were generated after positive selection with G418. BALB/cA Jcl-nu/nu mice (n=5) were injected subcutaneously with 1 × 10^5^ mock B16 cells (a), cells stably expressing Myc-tagged Tle3 (1-782) (b), or cells stably expressing Myc-tagged Tle3 (1-140) (c). Representative photograph of a mouse 3 weeks after injection with B16 cells (E). The volume of resected tumors was quantified. The data are expressed as the mean ± SD (n = 5). ^*^, p < 0.05 (F).

TLE3 usually requires interaction with HDACs via the TLE3 GP domain to function as a co-repressor for the repression of several different transcription factors [15]. Furthermore, several studies have demonstrated that HDAC inhibitors repress proliferation of malignant melanoma [7–9]. We thus examined whether HDACs are involved in augmenting the effects of Tle3 on the proliferation of B16 cells. Using a GST pulldown assay, we first confirmed that Tle3 does indeed bind directly to Hdac1 (Supplementary Figure 3). Consistent with a role for HDACs in melanoma proliferation, treatment of B16 cells with trichostatin A (TSA), an HDAC inhibitor, resulted in a reduction of proliferation (Figure 6A). This effect could also be observed with two other HDAC inhibitors – Apicidin and M344 (Supplementary Figure 2A and 2B). TSA, Apicidin, and M344 were also able to suppress proliferation in HMV-II cells (Supplementary Figure 2C), and COLO679 cells (Supplementary Figure 2D). TSA and Apicidin also eliminated the positive effect of Tle3 overexpression on B16 cell proliferation (Figure 6A and Supplementary Figure 2E), suggesting that Tle3 functions via a HDAC-dependent mechanism. Tle3 (1-140), a truncated mutant of Tle3 lacking the HDAC-binding GP domain, failed to increase the expression of CyclinD1, and proliferation (Figure 6B-6D). Moreover, the size of tumors derived from the subcutaneous injection of B16 stably overexpressing Myc tagged-Tle3 (1-140) cells were not larger than that of control tumors (Figure 6E and 6F). Altogether, these data suggest that HDACs are required for the TLE3’s ability to increase proliferation of melanoma.

## DISCUSSION

The Groucho/TLE proteins are a family of transcriptional co-factors implicated in the regulation of cell proliferation, differentiation and cell-fate events in multiple tissues. In mesenchymal tissues, we and others have previously demonstrated that under physiological conditions TLE3 regulates proliferation and differentiation of adipocytes [20, 21], osteoblasts [21] and skeletal muscle cells [12]. Here in this study, we have extended our understanding of TLE3 function by exploring its role in the pathological proliferation of neural crest-derived melanocyte cancer cells. More specifically, we show that the TLE3 is enriched during the malignant alteration of melanocytes in human patient tumor samples, and we further present evidence that Tle3 promotes proliferation in murine B16 and human HMV-II melanoma cells by an HDAC activity-dependent mechanism. Finally, we demonstrated the clinical relevancy of Tle3 as a therapeutic target by showing that Tle3 knockdown led to a significant reduction in tumor size *in vivo*.

Consistent with a role for TLE3 in melanoma development, our analysis of gene expression data from patient samples showed that TLE3 mRNA was highly enriched in tumor samples compared to normal skin or benign skin nevi samples. A correlation between elevated TLE family expression and tumorigenesis was first observed in cervical squamous metaplasias and carcinomas [22]. Since then, TLE2 and TLE3 have also been shown to be induced during the malignant progression of meningiomas [23]. More recently, an alternatively spliced isoform of TLE3 was found to be significantly enriched in prostate tumors [24]. Taken together, dysregulation of TLE3 expression appears to have a major impact on tumorigenesis. Our data also demonstrates that Tle3 has a positive effect on the proliferation of melanoma cells. Overexpression of Tle3 led to an increase in the proliferation of B16 cells *in vitro* and *in vivo* whereas Tle3 knockdown decreased proliferation. This data is in agreement with our previous study in skeletal muscle where TLE3 expression promoted the expansion and proliferation of satellite cells [12]. TLE family proteins play multiple and sometimes opposing roles on cell proliferation. Although several studies have shown a correlation in TLE protein expression and tumor progression, few have demonstrated causality by manipulating TLE levels. Forced expression of TLE1 in embryonic fibroblasts promotes cell growth and transformation [25] and similarly, TLE1 transgenic mice develop lung tumors with increased alveolar epithelial proliferation [26]. In acute myeloid lymphoma, loss of TLE1 or TLE4 increases cell proliferation suggesting that under some conditions TLE proteins may act as tumor suppressors [27]. Our data not only shows an enrichment of TLE3 in melanoma, but also shows that knockdown of TLE3 can reduce cell proliferation and tumor growth thus identifying a potential therapeutic target.

The mechanism by which TLE3 affects melanoma proliferation remains unknown although our data strongly suggests that it involves the modulation of HDAC activity. TLE3 does not have DNA binding domain and is thus not expected to bind DNA on its own. Instead, TLE3, along with HDACs, are recruited to DNA by multiple transcription factors where they subsequently reduce the activity of several targets [11]. Our data demonstrates that TLE3 can indeed bind to HDAC1 and moreover inhibition of HDAC activity prevented the promotion of cell proliferation by TLE3. HDAC inhibitors have been shown to have anti-proliferative effects on malignant melanoma cells [7–9]. HDAC inhibitors induce the expression of cyclin dependent kinase inhibitors (CK1) p21 by increasing the acetylation of chromatin at the gene promoter region. High levels of p21 are associated with a G1/S phase cell cycle arrest due to the inhibition of CDK2 by p21 [8, 28]. In preliminary experiments, we have observed that overexpression of Tle3 significantly reduced the expression of CK1 (p21) (Data not shown), suggesting that Tle3 along with HDACs may interact with and inhibit the transcription factor(s) that regulate the expression of CK1 (p21). Our data does not however determine which specific class of HDACs are involved in these mechanisms (Supplementary Figure 2). In addition, since TSA treatment reduced the expression of TLE3 (data not shown) it is also possible that the suppressive effect of TSA on the proliferation of melanoma is due to direct HDAC repression of TLE3 expression. HDAC inhibitors also induce apoptosis in several kinds of malignant tumor cells [2]. Our preliminary data showed that over-expression of Tle3 reduced the number of apoptotic B16 cells induced by TSA treatment (data not shown), indicating that the reduction of apoptosis by Tle3 may be involved in the regulation of B16 tumor size by Tle3. Further experiments are needed to elucidate the exact role of TLE3 in the pathophysiology of melanoma.

Canonical Wnt (Wnt-β-catenin) signaling is mediated through β-catenin. Wnt-β-catenin is activated by the binding of secreted canonical Wnts, such as Wnt1 and Wnt3a to Lrp and Frizzled coreceptor complexes to stabilize intracellular pools of β-catenin and activate Tcf/Lef dependent transcription [29]. Although it is clear that β-catenin is critical during the early stages of melanocyte transformation [30], conflicting studies on the role of β-catenin in melanoma proliferation and metastasis have been published. Malignant melanoma patients with high levels of β-catenin have better prognosis [31, 32]. Wnt3a or small-molecule activators of β-catenin signaling reduce the proliferation in B16 melanoma cells and other human melanoma cell lines *in vitro*. B16 melanoma cells overexpressing Wnt3a also have decreased tumor size and metastasis when cells are implanted into mice [32, 24]. TLE family members, including TLE3, act as transcriptional co-repressors of canonical Wnt-β-catenin signaling via binding to the downstream effectors TCF/LEF and subsequently inhibiting Wnt target gene transcription [11, 33–35]. Indeed, in our *in vitro* experimental model, overexpression of Wnt3a decreased the proliferation of B16 cells and overexpression of Tle3 also repressed Wnt-β-catenin signaling induced by Wnt3a or a constitutively active form of β-catenin (Supplementary Figure 4). These data suggest that the suppression of Wnt-β-catenin signaling by Tle3 might be involved in the mechanism underlying the proliferative effect of Tle3 in B16 cells.

In conclusion, the reduction of TLE3 levels may provide a novel and beneficial method to control melanoma. Needless to say, it is important to examine the role of Tle3 not only in cell proliferation but also in cell apoptosis, invasion and distal metastasis to understand the full scope of melanoma especially *in vivo*.

## MATERIALS AND METHODS

### Microarray data mining

To examine *TLE3* expression in patient melanoma samples, dataset GSE3189 [19] was downloaded from the NCBI Gene Expression Omnibus (GEO) and analyzed.

### Mice

12 week-old male C57BL/6 and 12 week-old male BALB/cA Jcl-nu/nu mice were purchased from CLEA Japan Inc. (Tokyo, Japan). All mice were used in accordance with guidelines from the Kyushu Dental University Animal Care and Use Committee. All experiments were carried out with the approval of the Animal Use and Care Committee of the Kyushu Dental University (Approval number #17-23).

### B16 cells, transfection, and selection of G418 or blasticidin resistant clones

B16 cells (RCB1283) (MTA; RM87746), HMV-II cells (RCB0777) (MTA; RM87747), or COLO679 cells (RCB0989) (MTA; RM87746) were purchased from RIKEN BRC (RIKEN, Ibaragi, Japan). B16 cells were maintained in DMEM supplemented with 10% fetal bovine serum (FBS) and 2 mM L-Glutamine [36]. HMV-II cells were maintained in DMEM/Ham’s F-12 supplemented with10% FBS. COLO679 were maintained in RPMI Medium1640 supplemented with 20% FBS. B16 cells were transfected with Myc-tagged murine Tle3 (1-782), Myc-taged murine Tle3 (1- 140) [12], Myc-pcDEF3 empty vector (Control), shRNA against murine Tle3 (#1792), or shRNA against LacZ (Control) [29]. Cells transfected with Myc-pcDEF3 or Myc-pcDEF3 empty vector were treated with G418 (Roche, Basel, Switzerland) for 2 weeks until G418-resistant clones emerged. Cells transfected with shRNA against murine Tle3 (#1792) or LacZ (control), were treated with blasticidin (Wako, Osaka, Japan) to obtain blasticidin resistant clones.

### HDAC inhibitor treatment

B16 cells, HMV-II cells, or COLO678 cells were treated with trichostatin A (TSA) (Sigma Aldrich Chemicals, St. Louis, MO, USA), Apicidin (BioVison, Milpitas, CA, USA), M344 (BioVison, Milpitas, CA, USA), Sodium 4-Phenylbutyrate (BioVison, Milpitas, CA, USA), Splitomicin (BioVison, Milpitas, CA, USA), or Valproic Acid (VPA) (BioVison, Milpitas, CA, USA) at the indicated concentrations for 12 hours.

### B16 melanoma mouse model

Mice were injected subcutaneously with 1 × 10^5^ B16 cells in a 100 μL volume [37]. Tumor diameters were measured with calipers. Body weight and physiologic status were monitored daily.

### RNA isolation and quantitative real-time PCR

Total RNA was isolated from cells using FastGene™ RNA Basic Kit (Nippon Genetics, Tokyo, Japan) and then reverse-transcribed into cDNA using ReverTra Ace (Toyobo, Osaka, Japan). The cDNA was amplified by PCR using specific primers for murine *Tle3* (primer sequences: forward, agtctcgcctccattcctg; reverse, catctgcccatcagcactc), murine *Cyclin A2* (primer sequences: forward, cttggctgcaccaacagtaa; reverse, caaactcagttctcccaaaaaca), murine *Cyclin D1* (primer sequences: forward, tttctttccagagtcatcaagtgt; reverse, tgactccagaagggcttcaa), murine *Cyclin D2* (primer sequences: forward, tcccgactcctaagacccatc; reverse, taccagttcccactccagca), and *β-actin* (forward, aaggccaaccgtgaaaagat; reverse, gtggtacgaccagaggcatac). SYBR green-based quantitative real-time PCR was performed using PowerUp SYBR Green Master Mix (Thermo Fisher Scientific, Waltham, MA) with QuantStudio 3 system (Thermo Fisher Scientific). Values were normalized to *β-actin* using the 2^-ΔΔCt^ method [38].

### Immunohistochemistry analysis

Freshly isolated skin from C57BL/6 mice were immediately fixed in 4% paraformaldehyde in PBS and subsequently embedded in paraffin. Vertical sections, 6 to 8 μm thick, were deparaffinized in xylene and rehydrated with a graded series of ethanol concentrations. Sections were incubated at 4°C overnight with polyclonal anti-TLE3 antibody (Proteintech, Chicago, IL, USA), or normal Rabbit IgG (MBL, Aichi, Japan). After washing, sections were incubated for 1h with peroxidase-labeled secondary antibodies (Histofine Simple Stain)(Nichirei Biosciences, Tokyo, Japan). Diaminobenzidine (Histofine DAB-3S kit)(Nichirei Biosciences) served as the peroxidase substrate. ABZ-9000 (Keyence, Tokyo, Japan) microscope was used for these analyses.

### Immunocytochemistry analysis

B16 cells were incubated with primary antibodies at 4°C overnight following blocking/permeabilization with PBS containing 0.3% Triton X100 and 5% goat serum for 20 minutes at room temperature. The following antibodies were used for immunocytochemistry: polyclonal anti-TLE3 antibody (Proteintech), and CyclinD1 mouse monoclonal antibody (72-13G)(Santa Cruz, Santa Cruz, CA, USA). anti-Ki67 rabbit monoclonal antibody (ab92742, Abcam, Cambridge, UK). The target proteins were visualized using an Alexa 488- or Alexa 594-conjugated secondary antibody (Invitrogen, Carlsbad, CA, USA). ABZ-9000 (Keyence) microscope was used for these analyses. To visualize the cell nuclei, the cells were mounted with Hard Set Mounting Medium with DAPI (Vector laboratories, Burlingame, CA, USA) and to visualize the cellular skeleton, the cells were stained with Rhodamine Phalloidin (Thermo Fisher Scientific).

### Western blot analysis

The following antibodies were used for Western blot analysis: anti-TLE3 antibody (Proteintech), anti-cyclinD1 mouse monoclonal antibody (72-13G)(Santa Cruz), anti-Myc-tag polyclonal antibody (MLB), CyclinA2 rabbit polyclonal antibody (GTX103042, GeneTex, Irvine, CA), and anti-β-actin mouse monoclonal antibody (Sigma Aldrich Chemicals, St. Louis, MO, USA). The target proteins were detected using an anti-mouse or anti-rabbit IgG antibody conjugated with horseradish peroxidase (Cell signaling, Beverly, MA, USA) and visualized by ImmunoStar LD (Wako).

### Expression plasmids, shRNA, and siRNA

Myc-tagged murine TLE3 expression plasmid [12], C-terminally truncated forms of myc-tagged Tle3 plasmids [12], murine Wnt3a [32] and constitutively active form of murine β-catenin [39] were previously described. Super TOP flash luciferase reporter vector [40] was kindly provided by Dr. Randall Moon. Short hairpin RNA against murine Tle3 constructs (shTLE3) was designed using BLOCK-IT RNAi designer tool (Invitrogen), sense and antisense oligos were annealed and cloned into pcDNA 6.2-GW/miR [29]. The following shRNA oligos were used: control shRNA, TGCTGAAATCGCTGATTTGTGTAGTCGTTTTGGCCACTGACTGACGACTACACATCAGCGATTT; shRNA against Tle3, TGCTGTGCTGAGGCTGTCTTTCTCTT GTTTTGGCCACT GACTGACAAGAGAAACAGCC-TCAGCA. Only sense strands are shown [21, 29]. siRNA-1 against murine Tle3 (Stealth siRNA, MSS238514)(Thermo Fisher Scientific), siRNA-2 against murine Tle3 (Stealth siRNA, MSS2385136), siRNA-1 against human TLE3 (Stealth siRNA, HSS186348), or siRNA-2 against human TLE3 (Stealth siRNA, HSS110791) were transfected into B16 cells or HMV-II cells using Lipofectamine RNAiMAX Transfection Reagent (Thermo Fisher Scientific) according to the manufacturer’s Protocol.

### Cell proliferation assay

The proliferation of cells was assessed using a Cell Counting kit-8 (Dojindo, Kumamoto, Japan), according to the manufacturer’s Protocol [34].

### Luciferase assays

Luciferase assays were performed using Super TOPflash-luciferase reporter vector or phRL-SV40 (Promega, Madison, WI) with the Dual-Glo Luciferase Assay System (Promega)

### Statistical analysis

Comparisons were made using an unpaired ANOVA with Tukey-Kramer post-hoc test and Wilcoxon’s signed rank test. The results are shown as the mean ± S.D. The statistical significance is indicated as follows: ^**^, p < 0.01 and ^*^, p < 0.05.

## SUPPLEMENTARY MATERIALS FIGURES


