# Supplementary material for: Transducin-like enhancer of split 3 regulates proliferation of melanoma cells via histone deacetylase activity
Source: Oncotarget. 2019 Jan 8;10(3):404–14. doi: 10.18632/oncotarget.26552 (PMC6349449; doi:10.18632/oncotarget.26552)
Supplement: Supplementary file 1 [file oncotarget-10-404-s001.pdf]

Transducin-like enhancer of split 3 regulates proliferation of melanoma cells via histone deacetylase activity

SUPPLEMENTARY MATERIALS

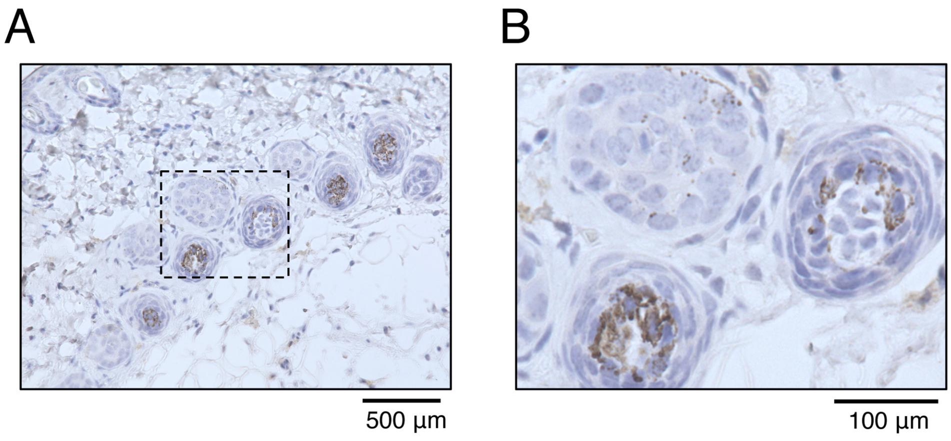

Supplementary Figure 1: Murine melanocytes in hair follicles were immunostained with normal rabbit IgG. Skin from 12-week-old C57BL/6J male mice was immunostained with Normal Rabbit IgG. The boxed area containing hair follicles **(A)** are shown magnified in Panel B. Scale bars correspond to 500 μm (A) and 100 μm **(B)** respectively. Representative images are shown (A and B).

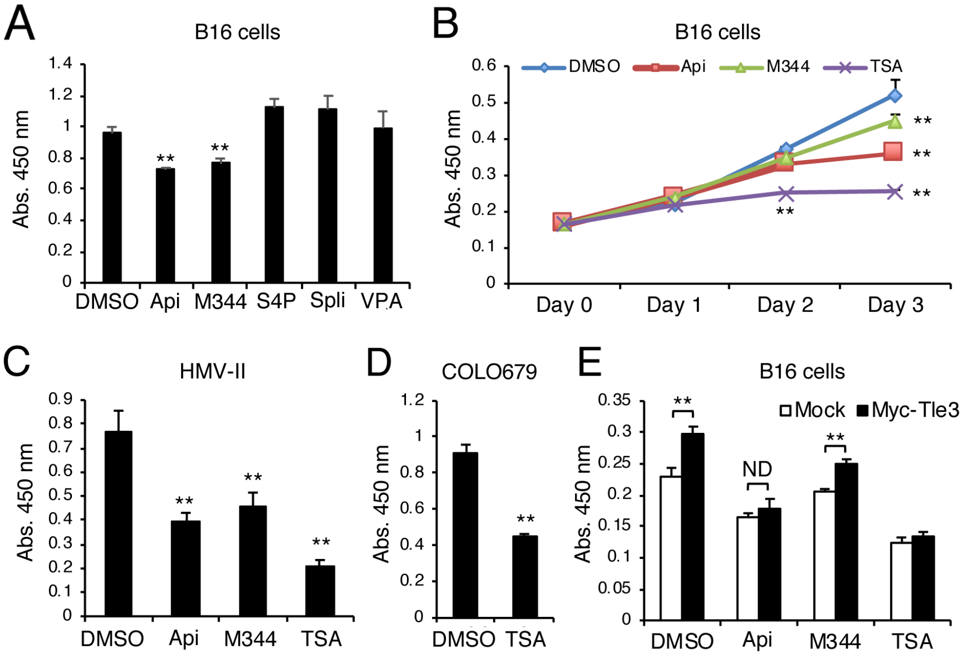

Supplementary Figure 2: Apicidin and M344 suppress the proliferation of melanoma cells. HDAC inhibitors such as Apicidin (Api), M344, Sodium 4-Phenylbutyrate (S4P), Splitomicin (Spli), Valproic Acid (VPA), or trichostatin A (TSA) were used at a 5 μM concentration. B16 cells were treated with DMSO, Api, M344, S4P, Spli, or VPA and cell proliferation was evaluated on day 2 by water-soluble tetrazolium salt (WST) assay and absorbance measurement at 450 nm **(A)**. In cells treated with Api, M344, or TSA, proliferation ability on day 2 and day 3 were decreased in comparison to control cells **(B)**. HMV-II **(C)** and COLO679 **(D)** cells were treated with DMSO, Api, M344, or TSA for 4 days (C and D). B16 cells were transiently transfected with empty vector (Mock) or Myc-tagged Tle3 and then treated with DMSO, Api, M344, or TSA. Cell proliferation was evaluated on day 2 by water-soluble tetrazolium salt (WST) assay and absorbance measured at 450 nm **(E)**. Data are expressed as the mean ± SD (n = 3). **, $p < 0.01$ versus control (A-E).

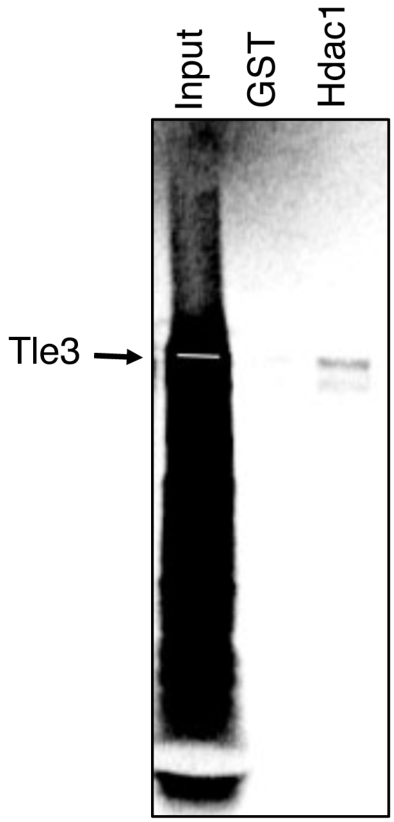

Supplementary Figure 3: Tle3 binds Hdac1. Murine Hdac1 (accession number; NM_008228) were amplified by standard PCR technique using PrimeSTAR HS DNA polymerase (Takara, Ohtsu, Japan) and inserted into GEX-4T1 GST-fusion vectors (GE Healthcare UK Ltd, Buckinghamshire, England). 35S methionine (PekinElmer, Pekin, China) labeled murine Tle3 protein was synthesized with an *in vitro* translation kit (Promega, Madison, WI). GST pulldown assay revealed that Tle3 directly binds to Hdac1 in a cell free system.

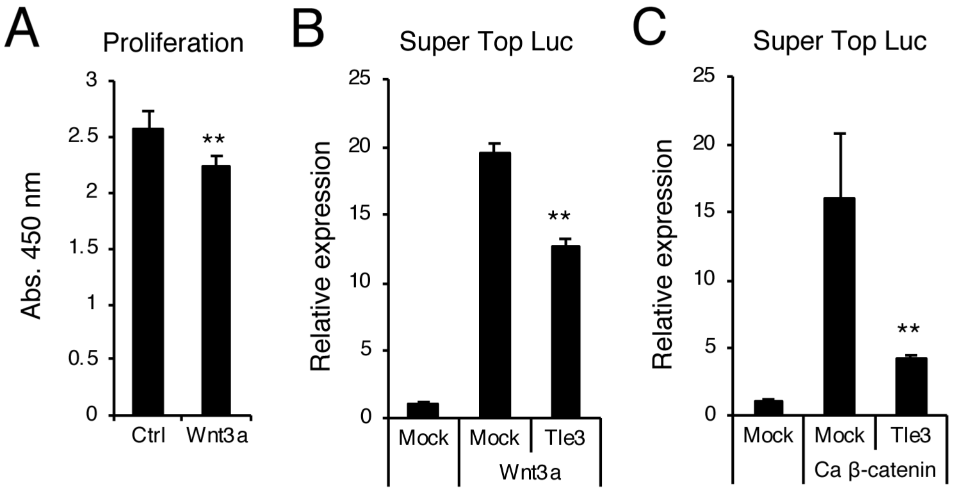

Supplementary Figure 4: Tle3 represses canonical Wnt signaling in B16 cells. B16 cells were transfected with control plasmid or Wnt3a. Cell proliferation on day 2 was assessed using cell counting kit-8 **(A)**. **(B and C)** B16 cells were transfected with Super TOP flash-luciferase reporter vector (Super Top Luc) together with Tle3, and Wnt3a (B) or a constitutively active form of β-catenin (C). Luciferase activity was determined 24 hours after transfection (B and C).
